# A flexible method for determining a biomarker positive population based on an efficacy target within a confirmatory study

**DOI:** 10.1186/1745-6215-16-S2-P133

**Published:** 2015-11-16

**Authors:** Chris Harbron

**Affiliations:** 1 Roche Pharmaceuticals, Welwyn Garden City, UK

## 

Precision medicine utilising biomarkers to identify those patients likely to obtain a benefit from receiving a particular treatment has the potential to deliver benefit across multiple stakeholders. These are dependent upon having a well-defined biomarker positive population, which for continuous biomarkers such as gene or protein expression includes defining a threshold or cut-off based upon observed clinical data. To obtain the desired precision in estimating such a cut-off frequently requires larger sample sizes than typically used in early phase studies.

A popular approach for investigating biomarker thresholds is the ‘minimum interaction p-value’ method (Jiang et al., Journal of the National Cancer Institute 2007). While this approach is flexible and relatively easy to implement, it operates independently of an efficacy target considered to be clinically meaningful.

We propose an approach that anchors the biomarker threshold to a clinically meaningful treatment effect. p-splines are used to flexibly model the efficacy-biomarker relationship in order to identify the threshold defining a biomarker positive population meeting a pre-defined efficacy target. The significance of this population is tested through permutation. The method is placed within a hierarchical split-alpha testing structure also permitting the selection of an all-comers population.

This innovative approach builds upon existing methods to define a biomarker cut-off and confirm efficacy within the same phase 3 trial. This method may lead to a viable path for more efficient clinical development programs and increased precision in threshold determination.

